# Early-Life Exposure to the Chinese Famine of 1959–1961 and Type 2 Diabetes in Adulthood: A Systematic Review and Meta-Analysis

**DOI:** 10.3390/nu14142855

**Published:** 2022-07-12

**Authors:** Chihua Li, L. H. Lumey

**Affiliations:** 1 Department of Epidemiology, Mailman School of Public Health, Columbia University, New York, NY 10032, USA; cl3106@cumc.columbia.edu; 2 Department of Endocrinology, Zhengzhou Central Hospital Affiliated to Zhengzhou University, Zhengzhou 450007, China

**Keywords:** famine, type 2 diabetes, methodological problems, age difference, famine intensity, study setting, confounding adjustment

## Abstract

Background: The fast-growing literature suggests that the Chinese famine of 1959–1961 drives current and future type 2 diabetes (T2D) epidemics in China. This conclusion may be premature, as many Chinese famine studies have major methodological problems. We examine these problems, demonstrate how they bias the study results, and formulate recommendations to improve the quality of future studies. Methods: We searched English and Chinese databases for studies that examined the relationship between prenatal exposure to the Chinese famine and adult T2D from inception to 8 February 2022. We extracted information on T2D cases and study populations of individuals born during the famine (famine births), before the famine (prefamine births), and after the famine (postfamine births). We used random-effects models to compare the odds of T2D in famine births to several control groups, including postfamine births, combined pre- and postfamine births, and prefamine births. We used meta-regressions to examine the impacts of age differences between comparison groups on famine effect estimates and the role of other characteristics, including participant sex, age, and T2D assessments; famine intensity; residence; and publication language. Potential sources of heterogeneity and study quality were also evaluated. Results: Twenty-three studies met our inclusion criteria. The sample sizes ranged from less than 300 to more than 360,000 participants. All studies defined the famine exposure based on the participants’ dates of birth, and 18 studies compared famine births and postfamine births to estimate famine effects on T2D. The famine and postfamine births had an age difference of three years or more in all studies. The estimates of the famine effect varied by the selection of controls. Using postfamine births as controls, the OR for T2D among famine births was 1.50 (95% CI 1.34–1.68); using combined pre- and postfamine births as controls, the OR was 1.12 (95% CI 1.02–1.24); using prefamine births as controls, the OR was 0.89 (95% CI 0.79–1.00). The meta-regressions further showed that the famine effect estimates increased by over 1.05 times with each one-year increase in ignored age differences between famine births and controls. Other newly identified methodological problems included the poorly assessed famine intensity, unsuitable study settings for famine research, and poor confounding adjustment. Interpretation: The current estimates of a positive relationship between prenatal exposure to the Chinese famine and adult T2D are mainly driven by uncontrolled age differences between famine births and postfamine births. Studies with more rigorous methods, including age-balanced controls and robust famine intensity measures, are needed to quantify to what extent the famine exposure is related to current T2D patterns in China.

## 1. Introduction

**Figure 1:**
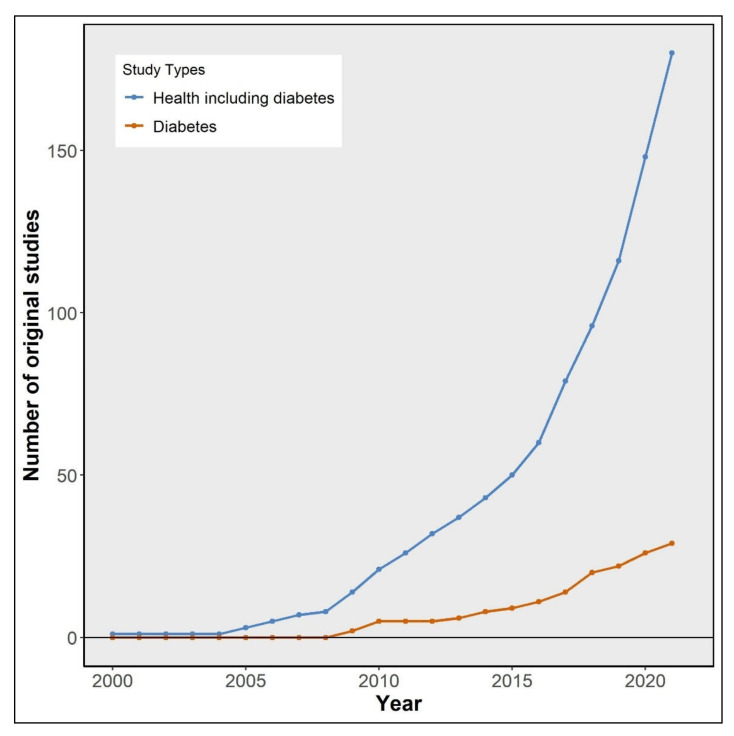
The cumulative number of original research articles on the Chinese famine and disease outcomes by year. The original research articles on the Chinese famine and disease outcomes included in this figure were identified using a similar search strategy as described in the methods section.

Famines in human history provide a unique opportunity to study how early-life environments may affect adult health [1,2]. In the past two decades, there has been an increasing interest in assessing the long-term health impact of prenatal exposure to the Great Chinese Famine of 1959–1961 (Chinese famine) [3,4,5]. As of December 2021, around 200 original research articles had been published relating the Chinese famine to a variety of adult diseases, including metabolic and cardiovascular conditions, reproductive health, psychological disorders, and many others (Figure 1). Type 2 diabetes (T2D) is the most widely examined disease, with over 30 articles to date. In addition, seven meta-analyses have been conducted to evaluate the long-term health impact of early-life exposure to the famine [6,7,8,9,10,11,12]. The studies and meta-analyses conclude that prenatal famine increases the risk of adult disease. Many reviews and commentaries also state that early-life exposure is a major driver of the current T2D epidemic in China and will be for T2D in future generations [3,13,14,15,16,17,18,19,20].

In 2017, we conducted the first systematic review and meta-analysis of long-term health after prenatal exposure to the Chinese famine. At the time, 36 studies had been published, of which seven reported on T2D [4]. We found that in nearly all studies, individuals born during the famine (famine births) were compared to individuals born after the famine (postfamine births), and that an increase in chronic disease in famine births was attributed to the famine. The older age of the famine births compared to postfamine controls creates a problem, as the risk of chronic disease increases with age and the apparent famine effect was no longer seen with age-balanced controls. Therefore, the design and analysis of Chinese famine studies needed further improvement before they could be used to accurately quantify the long-term impact of the famine. Since our 2017 review, over 150 additional Chinese famine health studies have been published, of which 24 are on T2D. Here, we conduct an updated systematic review and meta-analysis to examine if the selection of controls and the interpretation of study findings have changed after our initial review. Because of the large number of current health studies and the specific interest in long-term health effects of early-life famine exposure on T2D, we limited our review to this condition. We further examine additional study characteristics that may affect reported health outcomes (other than the age difference between comparison groups), perform quality assessments of all studies, and provide specific recommendations for future research.

## 2. Methods

### 2.1. Search Strategy and Study Selection

We followed the Preferred Reporting Items for Systematic Reviews and Meta-Analyses (PRISMA) guidelines (Supplementary Table S1) [21]. The study protocol is presented in Supplementary Text S1. The review was registered on Research Registry (UIN reviewregistry1352). Five electronic English- and Chinese-language databases were searched for Chinese famine studies on T2D outcomes from inception to February 8th, 2022: PubMed, EMBASE, Web of Science, Wanfang Data, and the Chinese National Knowledge Infrastructure (CNKI). The following broad search terms in English and Chinese were used to identify related studies, including journal articles, degree theses, and conference manuscripts: (([China OR Chinese] AND (famine OR undernutrition OR starvation OR malnutrition)) OR great leap forward OR great famine). Review articles and reference lists were screened for additional relevant studies.

Studies meeting the following criteria were included: (a) the study was original research; (b) the Chinese famine of 1959–1961 was the exposure of interest; (c) T2D, hyperglycemia, or increased blood glucose was the outcome of interest; and (d) clear information about the study design and results was provided. When the same or overlapping cohorts were reported in more than one study, we selected a representative study that either provided the most comprehensive information or had the largest sample size and excluded the others. Additional information on inclusion and exclusion criteria was included Supplementary Text S1. The full text of relevant studies was examined by both authors (C.L. and L.H.L.) to determine if they met the inclusion criteria. Discrepancies were resolved through consensus.

### 2.2. Data Extraction and Quality Assessment

We extracted author and publication information, study characteristics, the time window used to define different comparison groups, and tabular information on the number of T2D cases and the study population (Supplementary Text S1). A modified Newcastle–Ottawa scale was used to evaluate quality in three domains (study sample, design, and analysis) and contained eight items in total for each included study: sampling source, sample size, outcome assessment, exposure definition, control selection, famine intensity assessment, confounding adjustment, and statistical analysis (Supplementary Text S2) [22]. The quality of each item was scored as ‘good’ (2), ‘fair’ (1) or ‘poor’ (0), and a total score was calculated (range: 0–16). A study with a total score of over 10 was classified as of ‘good’ quality. Two reviewers (C.L. and L.H.L.) appraised each study independently, and discrepancies were resolved through consensus.

### 2.3. Statistical Analysis

Participants born during the Chinese famine of 1959–1961 were classified as famine births (prenatally exposed) in all studies; participants born after the famine were defined as postfamine births; participants born before the famine were defined as prefamine births. Some studies did not recruit prefamine births. In studies that included both prefamine births and postfamine births, the two groups were further combined. Therefore, three groups could be used as controls: postfamine births, prefamine births, and the combined pre- and postfamine births. For each study, the age difference between the famine births and available control groups was calculated.

The *meta* and *metafor* packages in R 4.1.0 were used to perform the meta-analysis. To assess how study results might change based on the choice of controls, odds ratios (ORs) and 95% confidence intervals (CIs) for T2D were calculated for each study by comparing famine births to the available control groups. A fixed-effect (Mantel–Haenszel) model and random-effects (DerSimonian–Laird) models were used to obtain summary effect estimates (ORs and 95% CIs) [23]. The I^2^ statistic was used to estimate the percentage of heterogeneity across reports, and I^2^ > 60% was used as a cut-off for substantial heterogeneity. Studies that did not report on the number of T2D cases and study population were not included in the meta-analysis. To examine the influence of single studies on the meta-analysis results, a leave-one-out analysis was conducted by omitting one study at the time and then repeating the meta-analysis. To identify potential study characteristics influencing the results, a meta-regression and subgroup analysis were performed stratified by age differences between famine births and controls, sex, mean age at the time of the survey, T2D measurement type, reported famine intensity, urban or rural residence, and publication language [24,25]. The publication bias was assessed using funnel plots and Egger’s regression test [26,27].

## 3. Results

### 3.1. Study Characteristics

**Figure 2:**
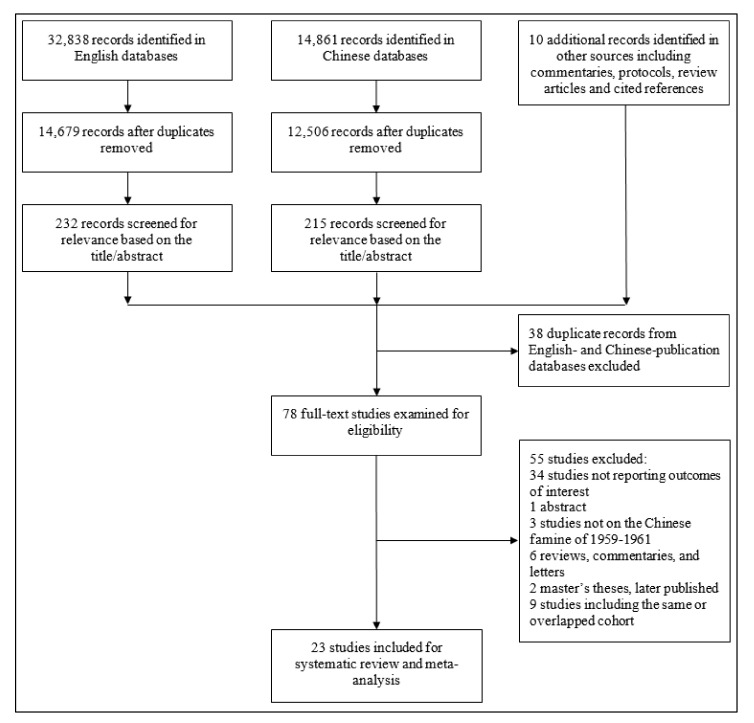
Flow diagram of study inclusion.

We identified 47,709 records from database searches and other sources (Figure 2). After removing duplicates and screening the title and abstract of each record, 78 studies were selected for full-text review. The full-text review identified 32 original studies relating the Chinese famine to T2D. Fourteen studies used identical or overlapping data sources from the Kailuan Group Health Examination [28,29], the China Kadoorie Biobank (CKB) [30,31], the China National Nutrition and Health Survey (CNNHS) 2010–2012 [32,33], the Survey on Prevalence in East China for Metabolic Diseases and Risk Factors Cohort (SPECT) [34,35,36], or the China Health and Retirement Longitudinal Studies (CHARLS) [37,38,39,40,41]. From these 14 studies, five studies were selected as the representative studies [28,30,33,34,38], and the other nine were excluded. Therefore, 23 Chinese famine studies on T2D were included for this review [28,30,33,34,38,42,43,44,45,46,47,48,49,50,51,52,53,54,55,56,57,58,59].

Table 1 summarizes the following characteristics of the included studies: authors, language, data source, outcome assessment, control selection, and reported results. Fourteen studies were in English, and the remainder were in Chinese. Eighteen studies used only postfamine births as controls, and five studies combined pre- and postfamine births as controls. The exact year and month of birth used to define famine births, prefamine births, and postfamine births in each study is shown in Supplementary Figure S1. Most studies used American Diabetes Association (ADA) or World Health Organization (WHO) definitions to classify T2D. The included studies show a 1.2- to 2-fold increase in the odds of T2D for famine births compared to controls, except for one study reporting a 5.7-fold increase (Study #17) [53].

Additional study information on the study design, sampling methods, sample size, famine intensity measurements, analytical methods, and covariate adjustments is summarized in Supplementary Table S2. Most studies were cross-sectional, except for two studies that followed participants’ T2D rates over time (Study #13 and 18) [30,55]. Eight studies adopted hospital- or corporation-based convenience sampling [28,42,43,44,47,48,49,58], and other studies used systematic sampling at both the regional and national levels. The sample size varied from less than 300 to more than 360,000 participants. Seven studies measured famine intensity based on either excess mortality rates or grain production in the 1950s–1960s [33,38,45,48,49,50,51]. Most studies used logistic regression to analyze the data. Three studies did not perform any covariate adjustment [42,44,48], and the remainder showed large variations in the covariates selected for confounding adjustment.

### 3.2. Age Differences Comparing Famine Births to Different Control Groups

**Figure 3:**
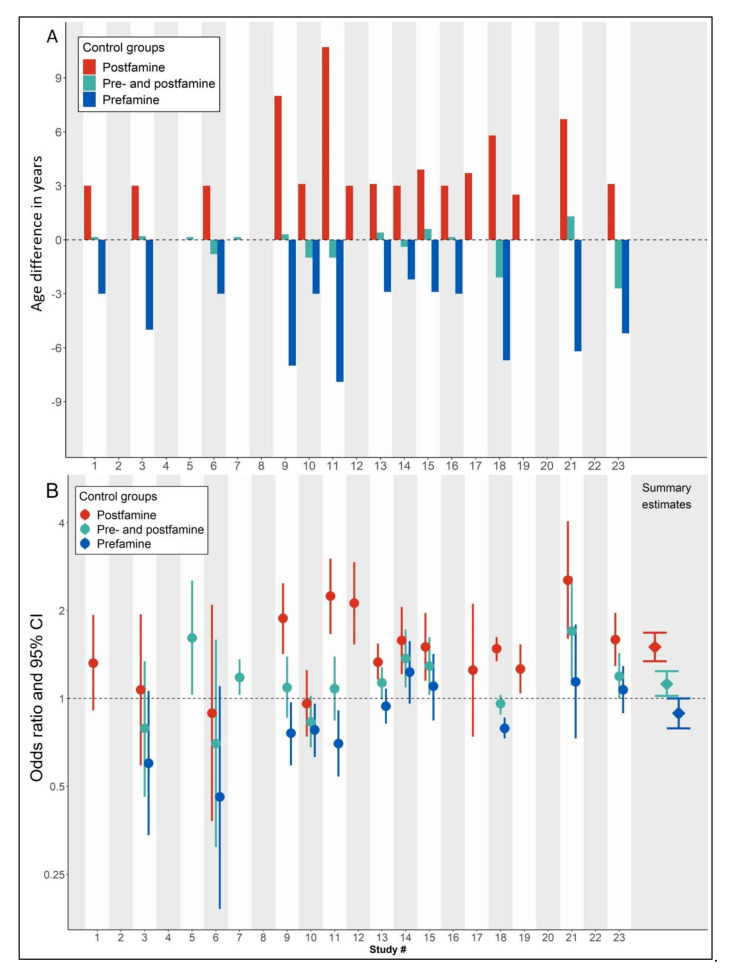
Age differences and effect estimates comparing the famine birth group to different control groups. (**A**) Age differences comparing famine births to different control groups, including the postfamine births, prefamine births, and combined pre- and postfamine births. (**B**) Effect estimates comparing famine births to different control groups. Odds ratios were calculated based on random-effects models and numbers of T2D cases and populations at risk. Summary estimates were generated based on the random-effects model.

We compared the mean age at the time of the survey of prefamine births, famine births, postfamine births, and the combined pre- and postfamine births across studies (Supplementary Table S3). Five studies (Study #2, 4, 8, 20, 22) did not provide any age information [43,44,48,56,58]. Four studies (Study # 2,12,17,19) did not recruit prefamine births [33,43,51,53]. Figure 3A shows the age differences of famine births and the three control groups. The age difference was three years or more when comparing famine births to either postfamine or prefamine births and one year or less comparing famine births to combined pre- and postfamine births, except in three studies (studies 18, 21 and 23) [55,57,59]. This shows that the age difference between famine births and controls will be smallest when combining prefamine and postfamine births as controls.

### 3.3. Different Study Findings Comparing Famine Births to Different Control Groups

We compared the effect size (ORs for T2D) of the contrast between famine births and each of the three control groups. Six of 23 studies did not provide any information about the number of T2D cases or any comparison group [43,44,48,54,56,58]. Figure 3B shows the ORs for the contrast in individual studies and also in summary effects comparing the famine births to either postfamine, combined pre- and postfamine, or prefamine births. Using postfamine controls, most studies show increased odds of T2D among famine births; using combined pre- and postfamine controls, most studies show no changes in odds of T2D; using prefamine controls, studies show either no relation or even decreased odds of T2D.

Further details on all individual studies as used in the meta-analysis of famine effect contrasts using different control groups are provided in Supplementary Figure S2A–C. Substantial heterogeneity between studies (I^2^ between 64–69%) was observed, regardless of the choice of control group. The random-effects model comparing famine births with postfamine controls showed an increased odds of T2D (OR 1.50, 95% CI 1.34–1.68) (Supplementary Figure S2A). In contrast, comparing famine births to the combined pre- and postfamine births as controls showed no or only a marginally increase in T2D odds related to famine in most studies (OR 1.12, 95% CI 1.02–1.24) (Supplementary Figure S2B). Compared to prefamine births, famine births even showed an overall ‘protective effect’ (OR 0.89, 95% CI 0.79–1.00) (Supplementary Figure S2C). The leave-one-out analysis showed that the above meta-analysis results were robust by omitting one study at a time (Supplementary Figure S3A–C). Therefore, the direction and magnitude of the famine effects on T2D were highly sensitive to the selection of controls.

### 3.4. Meta-Regression of Famine Effect Estimates over Age Differences and Other Characteristics

**Figure 4:**
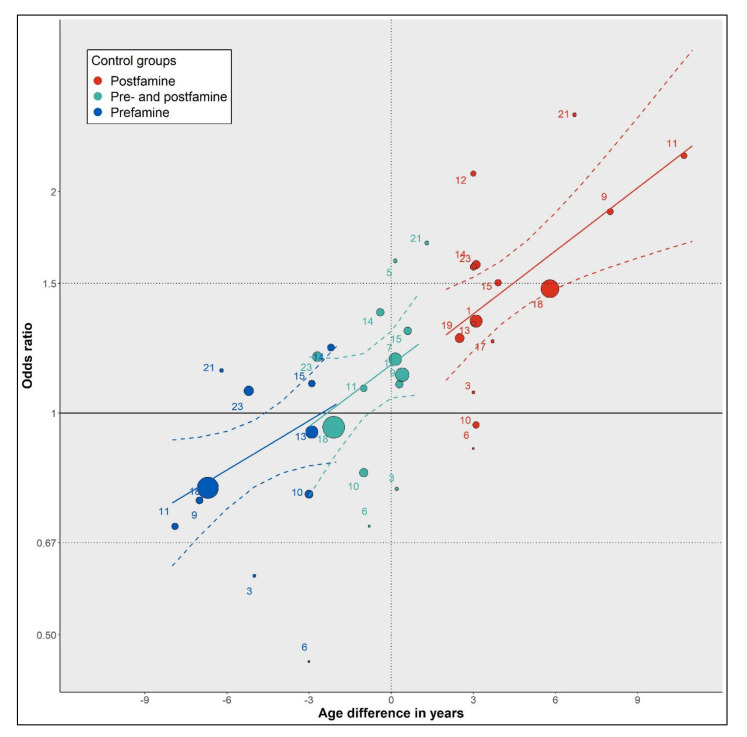
Meta-regression analysis of famine effect estimates over age differences between famine births and different control groups. The size of each dot is proportional to the weight of the study. The dashed colored lines represent the 95% CI for each meta-regression model.

We further examined the relationship between age differences and famine effect estimates using different control groups (Figure 4). Comparing famine births to postfamine births, the meta-regression showed that each one-year increase in age difference was associated with a 1.07-fold OR increase (95% CI 1.02–1.11); comparing famine births to combined pre- and postfamine births the OR increase was also 1.07-fold (95% CI 0.98–1.07); comparing famine births to prefamine births, the OR increase was 1.05-fold (95% CI 1.00–1.11). The famine effect estimates, therefore, show a consistent increase with increasing age differences between famine births and controls, irrespective of how these are defined.

Using the combined pre- and postfamine births as the control group, a further subgroup analysis was conducted to examine whether any other characteristics might modify the study findings. We further examined the relation within strata defined by sex (male, female, mixed), mean age at the time of the survey (<50 years, ≥50 years), T2D classification (WHO, ADA, ICD-10), reported famine intensity (severe, less severe, mixed), residence (urban, rural, mixed), and publication language (English, Chinese). We found no significant effect changes within any of the subgroups (random-effects model ORs between 0.96 and 1.25) (Table 2). Further details are provided in Supplementary Figure S4A–F. This confirms that the choice of controls and the age difference between famine births and controls within each control group are the most important drivers of estimated famine effects on T2D.

### 3.5. Quality Assessment

**Figure 5:**
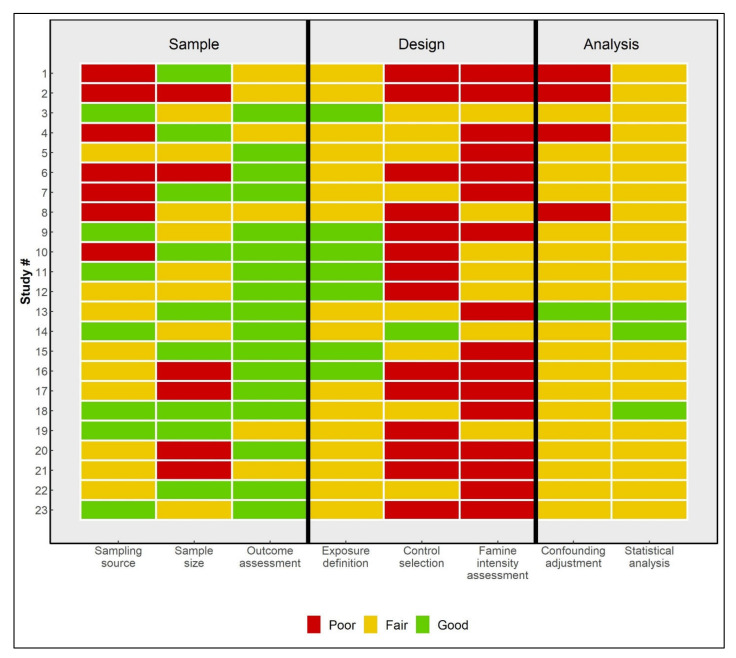
Quality assessments of the included studies. Study # is the same as in Table 1.

We examined small-study effects or publication bias of effect estimates comparing famine births to combined pre- and postfamine births. A visual inspection of the funnel plot and Egger’s regression test showed no plot asymmetry (Supplementary Figure S5) or significant test results. According to the quality assessment, most studies were of poor or moderate quality, with total scores ranging from 3 to 10, except for four studies that scored over 10 (studies 3, 13, 14, 18) (Figure 5) [30,38,45,55]. Most studies scored ‘good’ in the outcome assessment but ‘fair’ or ‘poor’ in the control selection and famine intensity assessment.

## 4. Discussion

In 2017, we noted that studies of T2D and other diseases in relation to early-life exposure to the Chinese famine had interpreted the increased prevalence of T2D and other diseases in famine births compared to postfamine controls as a famine effect, ignoring the age difference between the comparison groups. The current review examines changes in control selection and the interpretation of findings in T2D studies up to February 2022. Below, we further quantify the impacts of control selection and age differences between the comparison groups on the study results and comment on how famine intensity was assessed in the included studies, on the use of existing surveys, on covariate adjustments, and on the limitations of systematic reviews to date on the relation between the famine and T2D. We then provide recommendations on the design of future studies to more reliably estimate the impact of the Chinese famine on T2D.

### 4.1. Control Selection and Age Difference between Comparison Groups

Current Chinese famine studies continue to compare rates of T2D in famine births to T2D in postfamine controls to quantify the long-term impact of early-life famine exposure on T2D, ignoring that famine births are on average 3 years older than postfamine births and that T2D prevalence increases with age. This age difference explains the apparent famine effect reported by most current studies. Improvements in control selection are still needed to minimize the age difference with famine births.

The strong age effect on T2D in all studies is further illustrated by the meta-analysis and meta-regression. These approaches show the need to avoid significant age differences between famine births and the selected controls. Most studies to date used younger postfamine births as controls and could not differentiate between age effects and famine effects. Since the famine affected all provinces of China, it may be difficult to find unexposed controls with a similar age structure as the famine births [60,61,62]. Even combining pre- and postfamine births may not always generate an age-balanced control group if the number of prefamine and postfamine births is not approximately equal. Other analytical approaches to address this problem may then be needed, including difference-in-difference (DID) and age–period–cohort (APC) approaches. These strategies have been successfully applied in recent Chinese famine studies [37,38,63,64]. A shared characteristic of these studies, however, is that they all recruited prefamine, famine, and postfamine births from areas with different levels of famine intensity. Thereby, it was possible to both control for age differences and to examine a potential dose–response relationship between famine exposure severity and later disease.

The use of prefamine births as appropriate controls in Chinese famine studies has not yet been generally accepted for fear that early-childhood famine exposure may increase the odds of T2D or other diseases [34,50,55,57,65,66,67,68], perhaps also because of the findings of increased odds of T2D among prefamine births compared to postfamine births. These findings could again be misleading because the age difference between the comparison groups was at least 6 years and could even be up to 10 years [34,50,55,57]. Our exploration of CHARLS data shows no increased odds or risk of T2D in China among prefamine births when age is taken into consideration [69]. In other well-documented famines, the prefamine births in the Dutch Hunger Winter famine of 1944–1945 and the Ukraine Holomodor famine of 1932–1933 show no increase in T2D compared to the postfamine births [70,71,72]. At this point, it appears that the older age rather than famine exposure in childhood explains the T2D increases among prefamine births in China.

### 4.2. Famine Intensity

Most studies defined famine exposure by year or month of birth alone and ignored additional information on regional differences in famine intensity [4,5,73]. This provides only limited and possibly misleading information on famine intensity and is likely to result in misclassification of the famine exposure status. It is possible, however, to add ecological measures of famine intensity at the regional level as indicators of available foods at the individual level. This information can be useful to refine famine exposure because measures of individual energy intake at the time of the famine are not available [1,4]. As an example, regional mortality has been used as an ecological measure of famine intensity, using 50% excess mortality or more during the famine as the cut-off point to classify famine intensity as ‘severe’ or ‘less severe’ in studies of T2D [38,45,49,50] and other disease outcomes [68,74,75,76]. However, the use of a single cut-off point should be avoided, as this can lead to significant misclassification. By illustration, the provinces of Jilin (56.4% increase in mortality) and Anhui (474.9% increase in mortality) were accordingly both classified as ‘severe famine’ areas despite a nine-fold variation in excess mortality [45,77,78]. To be informative about possible dose–response effects, famine intensity should, therefore, be classified into at least three or four levels. Grain production records have also been used as regional measures of famine intensity [48,51,79]. This may not be appropriate, as grain production in China was not the major cause of the famine [80,81,82,83,84,85,86,87]. To better assess famine intensity at the local or regional level, relevant information from documents and studies in other disciplines including history, demography, and economics can also be examined to generate a severity grouping of three or four levels that is consistent across disciplines [69,88,89]. A robust indicator of famine intensity of this nature will facilitate the identification of potential dose–response effects and the comparison of results across studies.

### 4.3. Use of Existing Surveys

Most Chinese famine studies have been based on existing cross-sectional surveys or cohorts. These may not all be suitable for the reliable identification of famine effects because of missing information on the study sampling methods, study setting, and personal characteristics of study participants. For example, in many studies with convenience sampling, it was not possible to relate the participants to a well-defined study population of individuals with or without famine exposure [28,42,43,44,47,48,49]. This provides challenges to the interpretation of the study findings and the generalizability of outcomes to other populations. In the recruitment scheme of the available settings, the number of famine births tends to be much smaller than the number of prefamine births or postfamine births [60,62]. This compromises the study power and could be avoided by the oversampling of famine births in any study specifically designed to evaluate long-term famine effects. Regarding the personal characteristics that could help in the further interpretation of the study findings, important information on the place of birth and residence and familial socioeconomic status (SES) during the famine was not collected in many studies. Such information is important in view of the substantial rural–urban differences in famine intensity and the prevalence of T2D in China [34,54] and the important role of familial SES during the famine in influencing both famine exposure and T2D [90,91]. In the design of future famine studies, solutions have to be found to address the inherent limitations of existing surveys or cohorts.

### 4.4. Covariate Adjustments

Most T2D studies have attempted to address confounding factor via statistical adjustment for various combinations of covariates, but many such adjustments could be questioned. For instance, what could be the rationale to adjust for body size when estimating the association of famine exposure and T2D when it is not clear whether body size is an effect modifier or a mediator of this relationship [30,92,93,94,95]? If body size modifies the relationship between famine exposure and T2D, the analysis could be stratified by body size [30,95]; if body size is a mediator of this relationship, however, adjustment will lead to the underestimation of any famine effects on T2D [93,94]. Several studies performed adjustments for both the body mass index (BMI) and waist circumference in a single model, which may lead to problems of collinearity [28,30,50,53]. Smaller studies performed adjustments for a set of covariates that was large in relation to the number of participants, forcing multivariate regressions on variable combinations with many empty cells [45,47,51,53,57]. The covariate adjusted associations between famine exposure and T2D could differ substantially from the association without adjustments [30,45,53,94]. For instance, there is a need to question the famine effect in a study where the reported crude OR was 1.25 (95% CI: 0.74–2.11) and the adjusted OR was 5.71 (95% CI: 1.53–21.2) [53]. Such differences in crude and adjusted estimates have seldom been explored but could lead to further insights [94]. The use of causal knowledge and graphs including directed acyclic graphs (DAGs) in future studies could help guide the rationale and need for specific covariate adjustments [96,97].

### 4.5. Methodological Problems of Other Systematic Reviews to Date

Several other systematic reviews and meta-analyses have attempted to clarify the relationship between early-life famine exposure and disease outcomes [4,6,7,8,9,10,11,12,98]. On closer examination, these meta-analyses all pooled maximally adjusted T2D effect estimates comparing famine births to postfamine births without assessing the type, age, or number of selected controls [6,9,10]. All reviews reported a 1.4-fold increase in the odds for T2D for famine births compared to controls. While this is consistent with our current estimate using postfamine births as controls, we have demonstrated that age-adjusted controls are needed for an unconfounded estimate of famine effects. Failing to recognize important age differences between famine births and study controls and other methodological problems in Chinese famine studies as outlined studies will generate misleading results.

### 4.6. Recommendations for Future Chinese Famine Studies

Based on above the findings, we formulated recommendations for the design and analysis of future Chinese famine studies on both T2D and other diseases (Supplementary Table S4). In the sample and survey stage, it is important to recruit participants born before, during, and after the famine from regions with different levels of famine intensity and to collect key information for the purpose of famine research. In the design and analysis stage, it is important to use appropriate analytical approaches and to justify covariate adjustments via causal considerations and graphs. Most Chinese famine studies on other diseases have used similar data sources and analytical methods as the reviewed T2D studies and will have similar methodological problems as we discussed above. This shows the importance of addressing these methodological problems not only for T2D studies, but broadly across all Chinese famine studies of long-term outcomes.

### 4.7. Limitations

Our review also has some limitations. Because of variations in the design and methods of Chinese famine studies, the use of meta-analyses to estimate summary estimates may not be appropriate. In this review, however, a meta-analysis was used as a tool to explore how the control selection and age differences between comparison groups can lead to systematic differences in study outcomes. As we did not have access to the original data for most included studies, we were not able to answer some other important questions. For example, it is unclear why the adjusted associations were so different from the crude associations in some studies and if a dose–response relationship could be further established.

Considering the current heavy burden of T2D in China, it is necessary to examine early-life environmental factors that may have contributed to this epidemic. Most of the current Chinese famine studies have serious methodological shortcomings. Our recommendations to address these shortcomings should be considered in future studies to improve their quality and to generate more reliable estimates of famine effects on T2D and other diseases. These efforts will provide important evidence and recommendations for public health policies.

## Figures and Tables

**Table 1:** Main characteristics of Chinese famine studies on type 2 diabetes (T2D).

Study #	Authors	Language	Data Source	Outcome Assessment	Control Selection	Reported Famine Effect on T2D *
1	Liu et al., 2009 [42]	Chinese	Chongqing First Hospital Affiliated Health Examination Center, 2007	Fasting blood glucose	Post	Increased level of fasting blood glucose and prevalence of T2DM
2	Guan et al., 2009 [43]	Chinese	Chongqing Gangtie Group, 2009	Fasting blood glucose	Post	Increased level of fasting blood glucose
3	Li et al., 2010 [45]	English	China National Nutrition and Health Survey (CNNHS), 2002	WHO 1998	Post	ORs: 1.43 (0.53, 3.87) for severe famine areas; 0.41 (0.12, 1.35) for less severe famine areas
4	Li et al., 2010 [44]	Chinese	Chongqing First Hospital Affiliated Health Examination Center, 2010	Fasting blood glucose	Pre and Post	Increased level of fasting blood glucose
5	Zhang et al., 2010 [46]	Chinese	Tangshan Resident Study, 2009	ADA 1997	Pre and Post	OR: 1.69 (1.06, 2.69)
6	Zhao et al., 2013 [47]	Chinese	Anhui Medical University Affiliated Health Examination Center, 2011	WHO 1999	Post	RR: 0.91 (0.37, 2.23)
7	Li et al., 2014 [28]	Chinese	Kailuan Group, 2006–2007	WHO 1998	Pre and Post	OR: 1.22 (1.06, 1.40)
8	Zhang et al., 2014 [48]	Chinese	Bengbu First Hospital Affiliated Health Examination Center, 2011	Fasting blood glucose	Post	No increased level of fasting blood glucose
9	Wang et al., 2015 [34]	English	Survey on Prevalence in East China for Metabolic Diseases and Risk Factors Cohort (SPECT) in Shanghai, Jiangxi, Zhejiang, 2014	ADA 2014	Post	OR: 1.63 (1.13, 2.35)
10	Wang et al., 2016 [49]	English	Dongfengtongji Cohort (DFTJ), 2008	WHO 1998 and ADA 2010	Post	OR: 1.03 (0.77, 1.38) Same results using either WHO or ADA criteria
11	Wang et al., 2017 [50]	English	Survey on Prevalence in East China for Metabolic Diseases and Risk Factors cohort (SPECT) in Anhui, 2014	ADA 2014	Post	OR: 1.90 (1.12, 3.21) for severe famine areas
12	Li et al., 2017 [51]	English	Suihua Cohort, 2015	WHO 1999	Post	OR: 1.75 (1.20, 2.54)
13	Meng et al., 2018 [30]	English	China Kadoorie Biobank (CKB), 2004–2008	ICD-10: E12&14	Post ^#^	HR: 1.25 (1.07, 1.45)
14	Wang et al., 2018 [38]	English	China Health and Retirement Longitudinal Study (CHARLS), 2011–2012	ADA 2017	Pre and Post	OR: 1.37 (1.09, 1.72)
15	Zhang et al., 2018 [52]	English	Chronic Disease Survey of Jilin Province, 2012	WHO 1998	Post ^#^	OR: 1.51 (1.15, 1.98)
16	Zhou et al., 2018 [54]	English	Hefei City Resident Study, 2011–2012	WHO 2006	Post	RR: 0.72 (0.16, 3.33)
17	Liu et al., 2019 [53]	Chinese	Guangxi Zhuang Nationality Resident Study, 2017	ADA 2017	Post	OR: 5.71 (1.53, 21.2)
18	Lu et al., 2020 [55]	English	China Cardiometabolic Disease and Cancer Cohort (4C), 2011–2016	ADA 2017	Post ^#^	RR: 1.17 (1.05, 1.31)
19	Zhang et al., 2020 [33]	English	China National Nutrition and Health Survey (CNNHS), 2010–2012	WHO 1999	Post	OR: 1.31 (1.01, 1.70)
20	Qi et al., 2020 [56]	Chinese	Shanghai Jiading Community, 2018	WHO 1999	Post	ORs: 1.52 (1.07, 2.14) for men; 1.74 (1.22, 2.50) for women
21	Ning et al.2021 [57]	English	Qingdao Diabetes Prevention Programme, 2006–2009	WHO 2006	Post	RR: 2.15 (1.29, 3.60)
22	Zhang et al., 2022 [58]	English	YiduCloud Clinic Data, 1999–2018	Clinical records	Pre and Post	Increased prevalence of T2D among both males and females
23	Huo et al., 2022 [59]	English	Henan Rural Cohort Study	WHO 1998 and ADA 2009	Post	OR: 1.65 (1.29, 2.09)

Pre: prefamine births; Post: postfamine births; OR: odds ratio; RR: relative risk; HR: hazard ratio. * Famine effect estimate based on fully adjusted model. ^#^ Postfamine births used as controls in main analysis; combined pre- and postfamine births used as controls in sensitivity analysis.

**Table 2:** Subgroup analysis of effect estimates by selected characteristics comparing famine births to combined pre- and postfamine births.

	Fixed-Effect Model		Random-Effects Model	
	OR	95% CI	OR	95% CI
Sex				
Men	1.22	(1.11, 1.34)	1.22	(1.11, 1.34)
Women	1.12	(1.02, 1.23)	1.22	(1.02, 1.46)
Mixed *	0.96	(0.89, 1.03)	0.96	(0.89, 1.03)
Mean age at survey				
<50 years	1.21	(1.08, 1.37)	1.27	(0.98, 1.66)
≥50 years	1.04	(0.98, 1.10)	1.09	(0.98, 1.21)
T2D measurements				
WHO	1.12	(1.03, 1.22)	1.11	(0.94, 1.32)
ADA	1.02	(0.95, 1.09)	1.14	(0.96, 1.37)
ICD-10	1.13	(0.99, 1.28)	1.13	(0.99, 1.28)
Reported famine intensity				
Severe	1.24	(1.01, 1.53)	1.25	(1.00, 1.56)
Less severe	1.18	(1.07, 1.29)	1.18	(1.03, 1.34)
Mixed *	1.01	(0.95, 1.07)	1.07	(0.92, 1.24)
Residence				
Urban	1.07	(0.96, 1.20)	1.06	(0.79, 1.43)
Rural	1.19	(1.00, 1.43)	1.19	(1.00, 1.43)
Mixed *	1.05	(0.96, 1.20)	1.13	(1.00, 1.28)
Publication language				
English	1.04	(0.99, 1.10)	1.11	(0.99, 1.24)
Chinese	1.20	(1.05, 1.36)	1.21	(0.91, 1.62)

* Summary effect estimates of studies that did not report tabular information on the number of T2D cases and populations at risk by sex, famine intensity, and residence.

## Data Availability

The data used in this study are all presented in the main tables or supplementary materials. A code is available from the authors on request.

## Supplementary Materials

The following supporting information can be downloaded at: https://www.mdpi.com/article/10.3390/nu14142855/s1. Text S1. Study protocol and search results. Text S2. Quality Assessment Coding Criteria. Table S1. PRISMA checklist of items to include when reporting a systematic review and meta-analysis. Table S2. Additional characteristics of included Chinese famine studies on T2D Table S3. Mean age at the survey for different comparison groups. Table S4. Recommendation list for future Chinese famine studies. Figure S1. Exposure definition timing in Chinese famine studies. Figure S2A. Effect estimates of famine exposure on T2D comparing famine births with postfamine births; Figure S2B. Effect estimates of famine exposure on T2D comparing famine births with combined pre- and postfamine births; Figure S2C. Effect estimates of famine exposure on T2D comparing famine births with prefamine births. Figure S3A. Effect estimates of leave-one-out analysis comparing famine births with postfamine births; Figure S3B. Effect estimates of leave-one-out analysis comparing famine births with pre- and postfamine births combined; Figure S3C. Effect estimates of leave-one-out analysis comparing famine births with prefamine births. Figure S4A. Effect estimates of famine exposure on T2D after stratification by sex comparing famine births with combined pre- and postfamine births; Figure S4B. Effect estimates of famine exposure on T2D after stratification by mean age at the survey comparing famine births with combined pre- and postfamine births; Figure S4C. Effect estimates of famine exposure on T2D after stratification by T2D measurements comparing famine births with combined pre- and postfamine births; Figure S4D. Effect estimates of famine exposure on T2D after stratification by famine intensity comparing famine births with combined pre- and postfamine births; Figure S4E. Effect estimates of famine exposure on T2D after stratification by residence comparing famine births with combined pre- and postfamine births; Figure S4F. Effect estimates of famine exposure on T2D stratified by publication language comparing famine births with combined pre- and postfamine births. Figure S5. Funnel plot of effect estimates of famine exposure on T2D comparing famine births with combined pre- and postfamine births.
